# A bi-specific inhibitor targeting IL-17A and MMP-9 reduces invasion and motility in MDA-MB-231 cells

**DOI:** 10.18632/oncotarget.25526

**Published:** 2018-06-19

**Authors:** Dana Koslawsky, Marianna Zaretsky, Ron Alcalay, Ohad Mazor, Amir Aharoni, Niv Papo

**Affiliations:** ^1^ Department of Biotechnology Engineering, The National Institute of Biotechnology in the Negev, Ben-Gurion University of the Negev, Beer-Sheva, Israel; ^2^ Department of Life Sciences, The National Institute of Biotechnology in the Negev, Ben-Gurion University of the Negev, Beer-Sheva, Israel; ^3^ Department of Biochemistry and Molecular Genetics, Israel Institute for Biological Research, Ness-Ziona, Israel; ^4^ Department of Infectious Diseases, Israel Institute for Biological Research, Ness-Ziona, Israel

**Keywords:** cancer therapy, cytokines, matrix metalloproteinases, metastasis, drug design

## Abstract

The cytokine IL-17A is associated with the progression of various cancers, but little is known about the molecular cross-talk between IL-17A and other tumor-promoting factors. Previous studies have shown that the IL-17A-mediated invasion of breast cancer cells can be inhibited by selective antagonists of the matrix metalloproteinase 9 (MMP-9), suggesting that the cross-talk between IL-17A and MMP-9 may promote cancer invasiveness and metastasis. Here, we present a novel strategy for developing cancer therapeutics, based on the simultaneous binding and inhibition of both IL-17A and MMP-9. To this end, we use a bi-specific heterodimeric fusion protein, comprising a natural inhibitor of MMPs (N-TIMP2) fused with an engineered extracellular domain (V3) of the IL-17A receptor. We show that, as compared with the mono-specific inhibitors of IL-17A (V3) and MMP-9 (N-TIMP2), the engineered bi-specific fusion protein inhibits both MMP-9 activation and IL-17A-induced cytokine secretion from fibroblasts and exhibits a synergistic inhibition of both the migration and invasion of breast cancer cells. Our findings demonstrate, for the first time, that dual targeting of inflammatory (IL-17A) and extracellular matrix remodeling (MMP) pathways can potentially be used as a novel therapeutic approach against cancer. Moreover, the platform developed here for generating the bi-specific IL-17A/MMP-9 inhibitor can be utilized for generating bi-specific inhibitors for other cytokines and MMPs.

## INTRODUCTION

Patients with cancer often exhibit poor response to monospecific therapy [1], in large part due to the multifactorial nature of the disease [2]. It has been suggested that dual-targeting agents, i.e., molecules that can target and manipulate the activity of multiple targets simultaneously, are superior over monospecific agents for clinical applications because they offer improved binding affinity, avidity, potency, and selectivity [3]. Most attempts to develop such dual-targeting agents have focused on developing bi-specific antibodies, which can simultaneously bind two antigens—usually well-established targets for cancer therapy—to induce a beneficial synergistic effect [4–8]. However, bi-specific antibodies are difficult to assemble due to their natural dimerization, and they can activate the immune system to induce various side effects [2, 9–12].

Two families of protein that were shown to be important in promoting a variety of different cancers are matrix metalloproteinases (MMPs) and pro-inflammatory cytokines [13]. Of the MMPs, MMP-9 appears to be a particularly important target for cancer therapy, as it is involved in extracellular-matrix remodeling, plays a direct role in the expansion of tumor cells [14], promotes tumor metastasis [15], and its increased expression has been associated with various types of cancer [16–22] and cancer-related phenomena [23, 24]. Of the pro-inflammatory cytokines, IL-17A has recently been associated with the progression of various types of tumor [25–32] and is considered to be an important target for cancer therapy because its inhibition reduces cancer progression in animal models [33, 34]. For instance, inhibiting IL-17A at tumor sites significantly suppresses CD31, MMP9, and VEGF expression in the tumor [35], while activating its receptor (IL-17RA) promotes early tumor development [28, 36]. Several studies demonstrated a cross-talk between IL-17A and MMP-9, making these two proteins ideal for dual-inhibition, non-antibody cancer therapy. It was previously shown that IL-17A is associated with an increased expression of MMP-9, leading to enhanced cancer invasiveness and metastasis [31, 32, 37]. In addition, IL-17A stimulates the expression of the MMP-9 mRNA [38], and IL-17A-dependent invasion of breast cancer cells can be inhibited by MMP-9 inhibitors [32]. Furthermore, IL-17A was shown to promote the migration and invasion of cancer cells by up-regulating the expression of MMP-9 and down-regulating the expression of the tissue inhibitor of metalloproteinase 2 (TIMP2)—a natural inhibitor of MMPs—via the p38/NF-kB signaling pathway [36, 39]. Finally, chemotherapy (for example, FOLFOX) is known to increase the expression of IL-17A [40], and an IL-17A neutralizing antibody has been shown to enhance the therapeutic responsiveness of established colon tumors [40, 41], which are associated with high MMP-9 activity [42]. Similarly, in breast cancer, higher expression of IL-17 was linked with a greater probability for recurrence, greater chemotherapy resistance (to docetaxel), shorter disease-free survival rate, and a poorer prognosis [34]. These data suggest that chemotherapy induces the remodeling of the tumor microenvironment to support the tumor cellular hierarchy through secreted factors [40]. Since several chemotherapies have been shown to increase the expression of IL-17A, it would be beneficial to combine chemotherapies with IL-17A-neutralizing antibodies.

Such evidence for a cross-talk between IL-17A and MMP-9 suggest that their simultaneously targeting can be highly beneficial for inhibiting tumor progression in various types of cancer, including breast cancer, gastric cancer, cervical cancer, hepatocellular carcinoma, and lung cancer [13, 29, 32, 36, 39].

We recently developed an improved soluble IL-17RA, which can efficiently block IL-17A-induced cytokine secretion in a cell line and inhibits psoriasis plaque formation in a mouse model [43]. In addition, we developed a TIMP2 ligand, which targets MMP-9 and shows a potent inhibitory activity *in vitro* [44]. In the current study, we fused TIMP2 and the engineered soluble IL-17RA to develop a novel bi-specific inhibitor, which simultaneously targets both MMP-9 and IL-17A. We explored the biochemical properties of the mono- and bi-specific inhibitors and show that fusing the two individual inhibitors does not compromise their biochemical properties. Using cell-based assays, we found that the bi-specific inhibitor exhibits superior inhibition of cancer cell invasion and migration, as compared with the mono-specific inhibitors, either alone or together. Our approach for simultaneously targeting MMP-9 and IL-17A paves the way toward the development of bi-specific inhibitors targeting the MMPs and pro-inflammatory cytokines for the generation of novel cancer therapy agents [36, 39].

## RESULTS

### Construction and production of the bi-specific MMP-9/IL-17 inhibitor

We designed the construct of the bi-specific heterodimer, termed here HD_N-TIMP2,V3_, to contain two main components. The first component is N-TIMP2 – a discrete protein domain that folds independently of the C-terminal domain of TIMP2 [45] and is both necessary and sufficient for inhibiting the catalytic activity of all MMPs [46]. The second component is a soluble IL-17RA, termed V3, which contains the extracellular domain of the IL-17A receptor with five point mutations (Supplementary Figure 1) that increase its affinity towards IL-17A by 6-fold [43]. We conjugated the two components by using a peptide linker (Supplementary Figure 2), and then sub-cloned the bi-specific heterodimer into a mammalian vector, which contained the TIMP2 natural leader peptide at the N-terminus, fused with the human IgG1 Fc and a 6×His sequence at the C- terminus. Thus, the heterodimer comprised a free N-terminal, which is crucial for its activity because the N-domain of TIMP2 is responsible for the binding and inhibition of MMPs. Both HD_N-TIMP2,V3_ and V3 were produced in mammalian HEK293F cells, and N-TIMP2 was produced in *Pichia pastoris.* All proteins were purified by affinity column chromatography and gel filtration as soluble proteins (Supplementary Figure 3A), yielding highly pure (95%–98%) N-TIMP2, V3, and HD_N-TIMP2,V3_ (Supplementary Figure 3B). It was previously shown that the extracellular domain of many receptors is highly glycosylated, and that such glycosylation can significantly contribute to the conformation of the receptor and its binding to the target ligand [47]. Indeed, using SDS-PAGE, we found that the mobility of both the bi-specific heterodimer HD_N-TIMP2,V3_ and V3 reflects proteins with a much higher molecular weight than predicted based on the amino acid sequence, namely, ∼120 kDa instead of 76 kDa for HD_N-TIMP2,V3_ and ∼100 kDa instead of 60 kDa for V3 (Supplementary Figure 3C). The reduced mobility is probably due to multiple glycosylations, which, for N-TIMP2, do not affect its binding to MMPs [48, 49]. Glycosylation in V3 was shown previously [43], and, therefore, it was not surprising that HD_N-TIMP2,V3_ showed differences in the calculated versus the experimentally obtained molecular weight. Differences were also observed in the mass spectrometry analysis of the two proteins (Supplementary Figure 4): while the molecular weight of N-TIMP2 was the same as predicted based on the amino acid sequence, those of HD_N-TIMP2,V3_ and V3 were higher (namely, 93 kDa and 76 kDa, respectively) than the prediction. As expected, subtracting the molecular weight of V3 from that of HD_N-TIMP2,V3_, as determined by mass spectrometry, is the molecular weight of N-TIMP2.

### HD_N-TIMP2,V3_ binds simultaneously to both IL-17A and soluble MMP-9_CAT_

**Figure 1:**
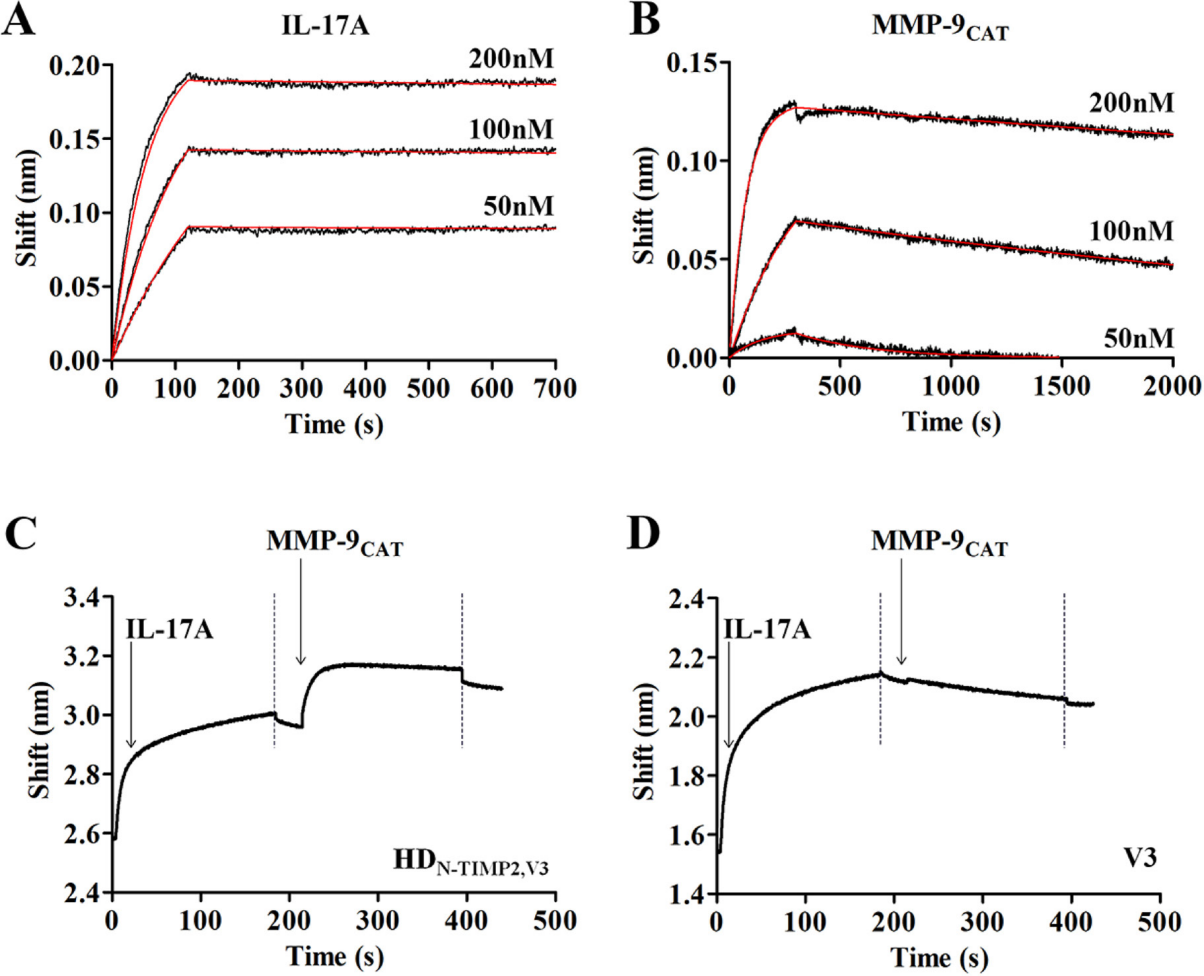
Binding of HD_N-TIMP2,V3_ to its ligands, IL-17A and MMP-9_CAT_ Real-time binding of HD_N-TIMP2,V3_ to its ligands was measured by using an Octet Red biolayer interferometry system. (**A**, **B**) HD_N-TIMP2,V3_ was immobilized on a Protein-A sensors and reacted for 300 s with increasing concentrations (black lines; from bottom to top: 50 nM; 100 nM, and 200 nM, as indicated) of either IL-17A (A) or MMP-9_CAT_ (B). The sensors were then immersed in a buffer for another 2500 s (dissociation phase). Red lines indicate curve fitting of a 1:1 binding model and used to determine the *K*_*D*_ values. (**C**) To evaluate the simultaneous binding of the two ligands, a Protein-A sensor coated with HD_N-TIMP2,V3_ was submerged in a solution containing IL-17A (leftmost arrow) and the wavelength interference was recorded. Following a short wash (leftmost dashed line), the sensor was immersed in solution containing MMP-9_CAT_ (rightmost arrow), followed by another washing step (rightmost dashed line). (**D**) As a control, a Protein-A sensor coated with V3 was submerged in a solution containing IL-17A and, following a short wash, the sensor was immersed in MMP-9_CAT,_ followed by another washing step.

After constructing the HD_N-TIMP2,V3_ heterodimer, we determined its ability to bind IL-17A and the soluble MMP-9_CAT_ (MMP-9 catalytic domain), using the Octet Red biolayer interferometry system [50], such that HD_N-TIMP2,V3_ was immobilized on a Protein-A Octet sensor, while its IL-17A binding sensogram was monitored at different concentrations of IL-17A. Then, the sensograms were fitted with a 1:1 binding model (Figure 1A) and the affinity constant (*K*_*D*_) was determined. This analysis revealed that the HD_N-TIMP2,V3_ heterodimer fully retains its ability to bind IL-17A, as its affinity (K_D_) toward IL-17A was 0.048 ± 0.006 nM (for comparison, the affinity of V3 toward IL-17A in the same system was 0.122 ± 0.004 nM). Similarly, HD_N-TIMP2,V3_ retained its ability to bind MMP-9_CAT_ (Figure 1B), with a *K*_*D*_ of 1.65 ± 0.02 nM.

To characterize the ability of HD_N-TIMP2,V3_ to simultaneously bind both IL-17A and soluble MMP-9_CAT_, the heterodimer was immobilized on a Protein-A Octet sensor and submerged in a well containing IL-17A, which resulted in a wavelength shift (Figure 1C). Following a short wash, the HD_N-TIMP2,V3_–IL-17A complex was immersed in a solution containing MMP-9_CAT_, which resulted in an additional shift, indicating that the heterodimer indeed binds these two ligands simultaneously. As a control, we immobilized the mono-specific V3 on a Protein-A Octet sensor and examined its interaction with IL-17A, which resulted in a wavelength shift (Figure 1D). In contrast to the heterodimer, submerging the V3–IL-17A complex in a solution containing MMP-9_CAT_ did not result in a wavelength shift (Figure 1D), indicating that the IL-17 and MMP-9_CAT_ ligands did not bind to each other. Taken together, these experiments demonstrate that the HD_N-TIMP2,V3_ heterodimer simultaneously binds both its ligands with high affinity.

### HD_N-TIMP2,V3_ inhibits the activity of MMP-9_CAT_ and IL-17A *in vitro*

**Figure 2:**
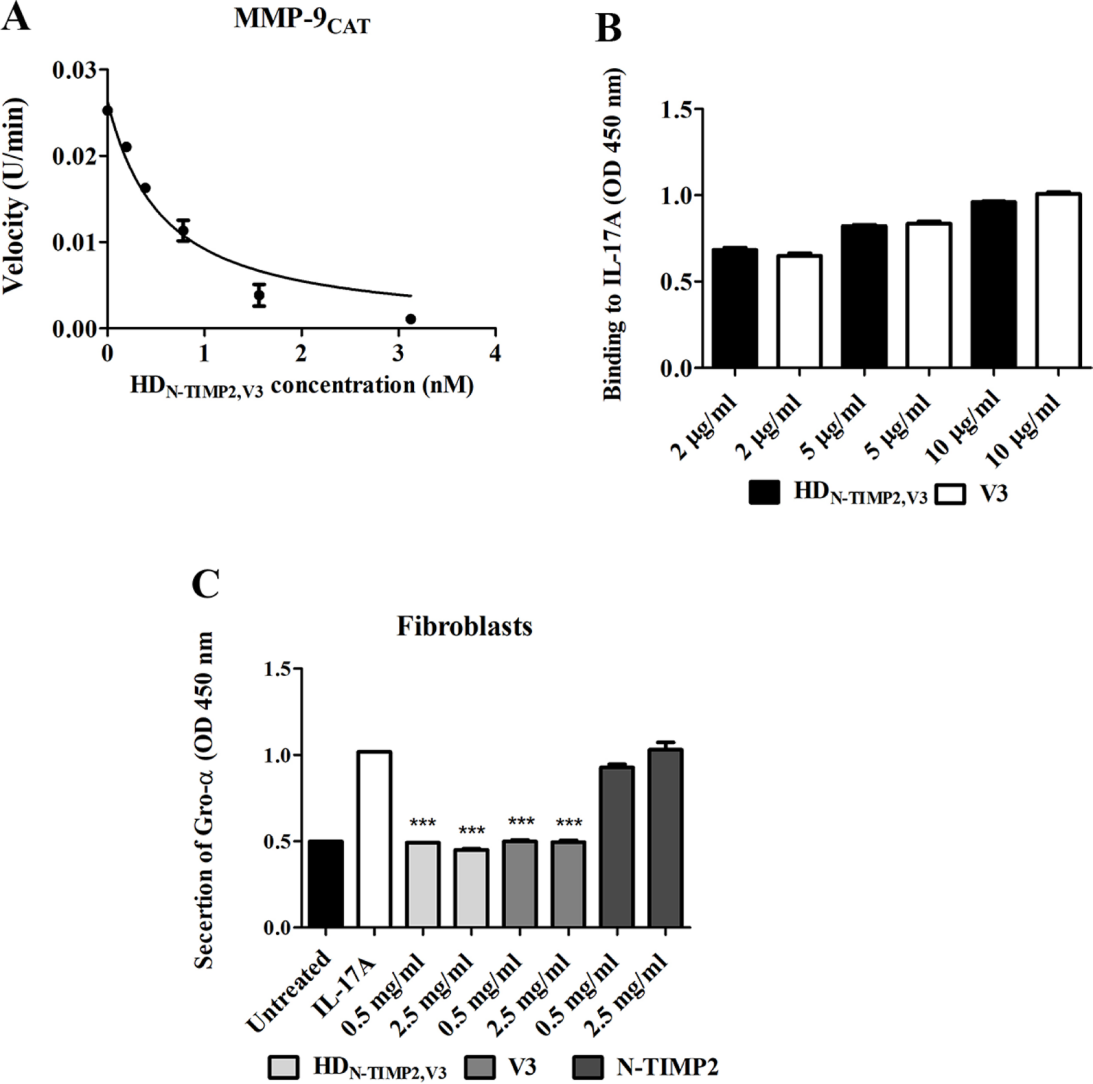
The inhibitory effect of HD_N-TIMP2,V3_, versus mono-specific controls, on the activity of MMP-9_CAT_ and IL-17A (**A**) Substrate degradation velocity of MMP9 CAT at different concentrations of HD_N-TIMP2,V3_ interacting with MMP-9_CAT_. Analyses were fitted to Morrison’s equation (see Eq. 1 in Materials and Methods) to obtain the *K*_*i*_ values. (**B**) An ELISA analysis of the binding of HD_N-TIMP2,V3_ and V3, at various concentrations, to immobilized IL-17A. (**C**) Fibroblast-based assay for the inhibition of IL-17A-induced Gro-α secretion. Cells were incubated with 10 ng/ml IL-17A and various concentrations of HD_N-TIMP2,V3_ or of the mono-specific controls (V3 or N-TIMP2). The undiluted cell supernatant was analyzed by ELISA to detect Gro-α levels. Data points indicate the mean (± SEM) of triplicate experiments. ^***^P < 0.005 (Student’s *t*-test, compared with cells treated with IL-17A alone).

To test whether the purified HD_N-TIMP2,V3_ inhibits the activity of MMP-9_CAT_ *in vitro*, we employed an enzymatic activity assay. This assay indicated that HD_N-TIMP2,V3_ inhibits the catalytic activity of MMP-9_CAT_ (Figure 2A) with a *K*_*i*_ of 99 ± 11 pM, which is similar to that previously reported for the inhibition of MMP-9_CAT_ by N-TIMP2 [*K*_*i*_ of 100 ± 8 pM [44]] and is in agreement with the *K*_*D*_ value of the binding of HD_N-TIMP2,V3_ to MMP-9_CAT_ (1.65 ± 0.03 nM). As expected, V3 alone did not inhibit the activity of MMP-9_CAT_ even at a concentration of 50 nM.

To test whether the purified HD_N-TIMP2,V3_ can bind IL-17A *in vitro*, we used an ELISA assay, which indicated that the binding to IL-17A is similar in both HD_N-TIMP2,V3_ and V3 (Figure 2B). Next, to examine the ability of HD_N-TIMP2,V3_ to inhibit the binding of IL-17A to IL-17RA in cells, we measured the inhibition of IL-17A-induced cytokine secretion in a cell-based assay, by measuring the secretion of Gro-α following the addition of IL-17A to human fibroblasts [51]. The addition of IL-17A (at 10 ng/ml, 0.32 µM) to the fibroblasts resulted in the secretion of more than 120 pg/ml Gro-α (Supplementary Figure 5). The addition of increasing concentrations of HD_N-TIMP2,V3_ together with 0.32 nM IL-17A to the fibroblasts led to a concentration-dependent inhibition of IL-17A-induced Gro-α secretion (Figure 2C), while the addition of 0.5 mg/ml (6.6 µM) HD_N-TIMP2,V3_ completely inhibited the IL-17A-induced Gro-α secretion (49.2% secretion, as compared with cells treated only with IL-17A; Figure 2C). HD_N-TIMP2,V3_ showed similar results to V3 (the positive control), namely, a 49.2% secretion as compared with 50%, respectively (Figure 2C), whereas N-TIMP2 (the negative control) did not inhibit Gro-α secretion (Figure 2C), as expected. Taken together, these experiments demonstrate that HD_N-TIMP2,V3_ efficiently inhibits the binding of IL-17A to its endogenous receptor, IL-17RA and inhibits MMP-9_CAT_ activity.

### HD_N-TIMP2,V3_, but not the mono-specific controls, strongly inhibits cancer cell invasion *in vitro*

**Figure 3:**
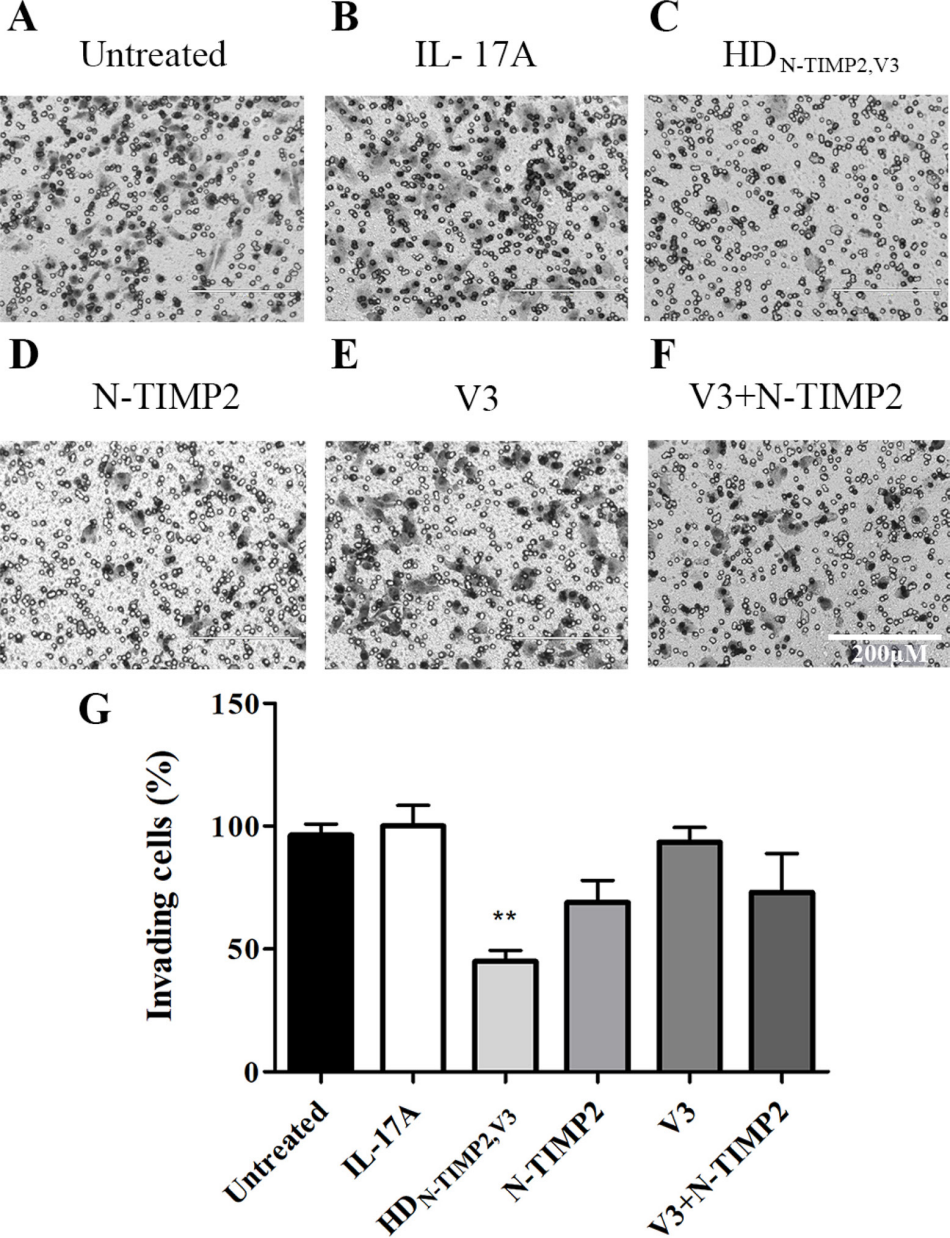
The inhibitory effect of HD_N-TIMP2,V3_, versus mono-specific controls, on the invasiveness of MDA-MB-231 cells MDA-MB-231 cells were treated with IL-17A, either alone or with HD_N-TIMP2,V3_ or the mono-specific controls, in Boyden chambers. The number of invading cells accumulating on the bottom of the membrane was counted. (**A**) Untreated cells. (**B**–**F**) Cells treated with IL-17A (10 ng/ml, 0.32 µM), either alone (B) or with 500 nM of HD_N-TIMP2,V3_ (**C**), N-TIMP2 (**D**), V3 (**E**), or N-TIMP2 + V3 (**F**). Scale bar in F applies to all images and represents 200 µm. (**G**) Quantification of invasive cells, normalized to cells treated with IL-17A alone. Bars represents the mean (± SEM) of triplicate experiments. ^**^P < 0.01 (Student’s *t*-test, compared with cells treated with IL-17A alone).

Previously described Matrigel invasion assays showed that the expression and catalytic activity of MMP-9 are essential for the invasiveness of different types of cancer cells [52–54], in particular MDA-MB-231 cells [52, 55]. Additionally, IL-17A was shown to promote the invasion of cancers cells, such as breast [56] and gastric [36, 38] cancer cells. As MDA-MB-231 cells express IL-17RA (Supplementary Figure 6), we evaluated the ability of HD_N-TIMP2,V3_, relative to that of the mono-specific controls, to inhibit the invasion of MDA-MB-231 cells in a Boyden chamber Matrigel invasion assay (Figure 3). MDA-MB-231 cells treated with IL-17A and 500 nM HD_N-TIMP2,V3_ showed a significantly lower invasiveness than untreated cells or cells treated with IL-17A (45% invasion, as compared with cells treated only with IL-17A; Figure 3A–3C, 3G), either alone or with N-TIMP2, V3 (69% and 93% invasion, as compared with cells treated only with IL-17A, respectively; Figure 3D, 3E, 3G), or a combination of N-TIMP2 and V3 (73% invasion, as compared with cells treated only with IL-17A; Figure 3F and 3G). These findings demonstrate the superior inhibitory effect of HD_N-TIMP2,V3_ over the mono-specific inhibitors (individually or combined), highlighting the importance of the cross-talk between MMP-9 and IL-17A for cell invasion.

### HD_N-TIMP2,V3_ inhibits cell migration in a scratch assay

**Figure 4:**
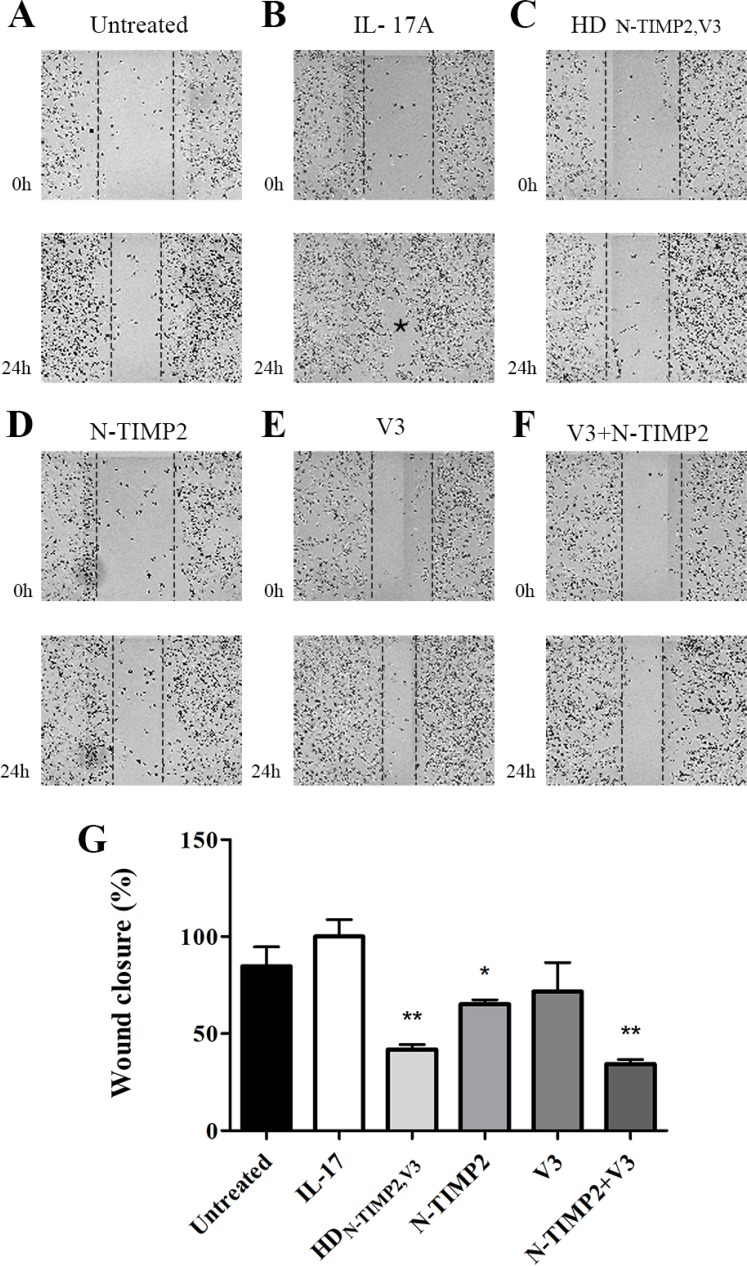
The inhibitory effect of HD_N-TIMP2,V3_, versus mono-specific controls, on the migration of MDA-MB-231 cells in a scratch assay MDA-MB-231 cells were scratched by removing a strip across the well. Then, the cells were treated with IL-17A, either alone or with HD_N-TIMP2,V3_ or the mono-specific controls, individually or together, for 24 h. The area free from cells was counted. (**A**) Untreated cells. (**B–F**) Cells treated with IL-17A (30 ng/ml, 0.95 µM), either alone (**B**) or with 10 nM HD_N-TIMP2,V3_ (**C**), N-TIMP2 (**D**), V3 (**E**), or N-TIMP2 + V3 (**F**). The asterisk in B represents a full migration of the cells to the scratched area. (**G**) Quantification of migrating cells, normalized to cells treated with IL-17A alone. Bars represents the mean (± SEM) of triplicate experiments. ^*^P < 0.05, ^**^P < 0.01 (Student’s *t*-test, compared with cells treated with IL-17A alone).

To further examine the ability of HD_N-TIMP2,V3_ (as compared with that of the mono-specific controls) to inhibit migration of MDA-MB-231 cells, we used a scratch assay. The extension of cell migration was quantified by estimating the percentage of recolonization of the scratch surface, 24 h after cell wounding (Figure 4). Treating the cells with 0.95 µM of IL-17A significantly promoted their migration, as compared with untreated cells (85% migration, as compared with 100% migration; Figure 4A, 4B and 4G). The addition of 10 nM HD_N-TIMP2,V3_ or a combination of N-TIMP2 and V3 (each at 10 nM) to IL-17A-treated cells considerably inhibited this migration (42% migration and 34% migration, respectively, with no significant difference between the two treatments; Figure 4C, 4F, 4G), whereas the addition of N-TIMP2 or V3 (each at 10 nM) alone had a smaller effect (65% and 72% migration, respectively; Figure 4D, 4E and 4G). Moreover, the inhibition of cell migration by HD_N-TIMP2,V3_ was dose-dependent (Supplementary Figure 7).

## DISCUSSION

We evaluated the therapeutic potential of targeting the MMP-9/IL-17A axis, which has been ascribed putative pathobiological roles in various types of cancer [32, 36, 37, 39]. To this end, we developed HD_N-TIMP2,V3_: a novel bi-specific heterodimer that successfully inhibits both MMP-9 activation and IL-17A-induced cytokine secretion to synergistically inhibit the invasion of breast cancer cells. Indeed, as the affinity of the N-TIMP2 and V3 domains of the heterodimer to their targets (MMP-9 and IL-17A, respectively) was not compromised, we attribute the improved ability of the heterodimer to inhibit cancer cell invasion to its ability to simultaneously bind both targets.

V3 is a soluble receptor that traps and inhibits IL-17A. IL-17A and MMP-9 (a target for N-TIMP) have been shown to cross-interact. It was previously shown that IL-17A is associated with an increased expression of MMP-9, leading to enhanced cancer invasiveness and metastasis [13, 29, 32, 36, 39]. In addition, IL-17A-dependent invasion of breast cancer cells can be inhibited by MMP-9 inhibitors [32]. Therefore, it is possible that simultaneous inhibition of both IL-17A and MMP-9 lead to synergism due to the cross-talk between the two pathways.

As such, our findings further highlight the importance of this cross-talk between MMP-9 and IL-17A for cancer cell invasion and migration (as was suggested previously [37]), and they may thus pave the way toward the development of a new generation of cancer inhibitors, based on the dual targeting of inflammatory and extracellular matrix remodeling pathways. We have previously developed a bi-specific inhibitor that targets both disintegrin and metalloproteinase 17 (ADAM17) and the cytokine TNF-like ligand 1A (TL1A) as a potential therapeutic approach for inflammatory bowel disease [57]. The bi-specific inhibitor exhibited high potency in cell-based assays, as it strongly bound ADAM17, displayed on the cell surface, to improve TL1A inhibition. Thus, a dual targeting of MMPs and pro-inflammatory cytokines can be highly beneficial as a therapeutic strategy for different complex diseases in which extracellular matrix remodeling is associated with an abnormal activation of the immune system.

A somewhat unexpected finding in our study was that the inhibitory effect of HD_N-TIMP2,V3_ was greater than that of a combination of its two mono-specific counterparts (N-TIMP2 and V3) in the invasion assay, whereas these two treatments had a similar effect in the migration assay. This finding may suggest that the degree of cross-talk between IL-17A and MMP-9 is lower in pathways involved in cancer cell migration than in cancer cell invasion. A hypothesis that will be addressed in future studies.

In addition to inhibiting cancer cell migration and invasion, bi-specific inhibitors that target both MMP-9 and IL-17 can increase the selectivity of treatment to those cancer cells that express both targets, in which the cross-reactivity and synergistic action of these targets strongly promote cancer cell invasion [36, 37], to improve tumor growth inhibition. Moreover, the improved potency of the bi-specific inhibitor, as compared with its mono-specific counterparts, can potentially reduce the side effects that may arise by inhibiting each target individually, as both MMPs and interleukins naturally play important roles in homeostasis, inflammation, and various important biological functions, such as wound healing [58], cell adhesion [59, 60], and cell differentiation [61].

In summary, our bi-specific inhibitor targeting MMP-9 and IL-17A can pave the way for the generation of additional bi-specific inhibitors targeting metalloproteinases and pro-inflammatory cytokines for targeting tumors in which the cross-talk between extracellular matrix remodeling and the immune system is a major driving force in cancer progression. The next logical step is therefore to directly test the therapeutic efficacy of the engineered bi-specific inhibitor, namely, its ability to inhibit tumor growth and reduce multiple metastases in various *in vivo* primary tumor and experimental metastasis models.

## MATERIALS AND METHODS

### Construction of the bi-specific HD_N-TIMP2,V3_ heterodimer

The pFUSE-hIgG1e3-Fc1 plasmid vector (InvivoGen, San Diego, CA) that contained the bi-specific heterodimer HD_N-TIMP2,V3_ was designed to include the following components: the N-TIMP2 natural leader peptide (residues 1–26, GenPept: NP_003246, NCBI), followed by N-TIMP2 (residues 1–127 [44]), a flexible linker (SGGGGSGGGGSGGGGS); the soluble extracellular domain of the IL-17A receptor, termed V3 [residues 1–288, including the mutations L10P, R109K, D123G, H156D, G244W, and A268V; [43]]; a human IgG1 Fc, used to increase the serum half-life, and a 6×His tag sequence at the C-terminus, used for protein purification and detection. The construct was obtained from Integrated DNA Technologies (Coralville, IA), and was amplified using a PCR reaction with a Phusion DNA polymerase (New England Biolabs, Ipswich, MA) with primers that were homologous to the pFUSE plasmid (forward-5′-CTG AGA TCA CCG GTG AAT TCA TGG GTG CAG CCG CAC GC-3′. reverse-5′-TAT CAT GTC TGG CCA GCT AGC ACT CAG TGA TGG TGA TGG TGA TGG ATA TC-3′). The PCR reaction comprised 10 ng of the construction gene, 1 μl of each primer, 0.4 μl Phusion DNA polymerase (New England Biolabs), 2 μl DNA polymerase buffer, 2 μl of a dNTP mix (10 mM), 1.2 μl MgCl_2_, and double deionized water, added to reach a total volume of 20 μl. The PCR conditions were: 95° C for 2 min, followed by 35 cycles at 95° C for 20 s, 56° C for 10 s, 70° C for 3 min, and a final elongation at 72° C for 5 min. The PCR product was cloned into the pFUSE vector by using the Gibson assembly method (New England Biolabs) according to the manufacturer’s protocol, followed by transformation into electro-competent *E. coli* cells, which were grown overnight on 25 μg/ml zeocin-LB agar plates. Then, 40 colonies were transferred to a 25 μg/ml zeocin-LB culture medium and grown overnight at 37° C. The plasmid was extracted from the bacteria using the HiYield plasmid mini kit (RBC Bioscience, Taiwan) and was sequenced in the genetics unit of the National Institute for Biotechnology in the Negev (NIBN), Ben-Gurion University of the Negev, Israel.

### Production and purification of recombinant proteins

The production of the bi-specific heterodimer was performed in mammalian HEK293F cells (Invitrogen, Carlsbad, CA), which were maintained in a FreeStyle™ 293 expression medium (Thermo Fisher Scientific, Waltham, MA). The construct was sub-cloned into the pFUSE mammalian vector, as described above. The HEK293F cells were transiently transformed with 1 µg/ml of DNA according to the FreeStyle 293 System manual, using the GeneTranIII transfection reagent (Biomiga, San Diego, CA). The supernatant was harvested after five days and the medium was then centrifuged for 10 min at 500 × g and filtered. The protein was purified from the filtered medium using a metal chelating chromatography with nickel-nitrilotriacetic acid-Sepharose beads (Invitrogen). The beads were washed with 50 mM Tris (pH 7.5), 100 mM NaCl, and 20 mM imidazole, and, following incubation with the medium, eluted with 50 mM Tris (pH 7.5), 100 mM NaCl, and 500 mM imidazole. The eluted protein was dialyzed and the buffer was exchanged to 50 mM Tris (pH 7.5), 100 mM NaCl, and 5 mM CaCl_2_, and was then concentrated with a 10 kDa cutoff Vivaspin (Vivaproducts, Littleton, MA). The protein was analyzed by western blotting, using an anti-human IgG antibody against Fc and an anti-6×His antibody against the His tag (both purchased from Abcam, Cambridge, UK), followed by anti-mouse alkaline phosphates (1:5000) (Jackson ImmunoResearch, West Grove, PA) and staining with 5-bromo-4-chloro-3-indolyl phosphate reagent (Sigma-Aldrich, Saint Louis, MO). N-TIMP2 and V3 were produced as previously described [43, 44]. The purified proteins were stored at 80° C until further analysis. To ensure protein purification, an SDS-PAGE analysis was conducted on a 15% polyacrylamide gel under reducing conditions; the bands were visualized by staining with Instant Blue (CBS Scientific, San Diego, CA) and a mass spectrometry analysis was performed on the purified proteins at the Ilse Katz Institute for Nanoscale Science and Technology, Ben-Gurion University of the Negev, Israel. Protein concentrations were determined by UV-Vis (absorbance at 280 nm), using a NanoDrop spectrophotometer (Thermo Fisher Scientific), with an extinction coefficient (ε_280_) of 99,820 M^-1^cm^-1^ for HD_N-TIMP2,V3_, 13,500 M^-1^cm^-1^ for N-TIMP2, and 80,995 M^-1^cm^-1^ for V3. The production process yielded an average of approximately 1 mg protein for each variant.

### MMP inhibition studies

The inhibitory activity of the HD_N-TIMP2,V3_ bi-specific heterodimer and of the N-TIMP2 and V3 mono**-**specific variants was tested against 0.0075 nM MMP-9_CAT_. MMP-9_CAT_ was incubated for 1 h at 37° C with either 0.4–25 nM HD_N-TIMP2,V3_, N-TIMP2 (0.4–25 nM), or V3 (0.4–25 nM) in a TCNB buffer containing 50 mM Tris (pH 7.5), 100 mM NaCl, 5 mM CaCl_2_, and 0.05% Brij. Then, the florigenic substrate Mca-Pro-Leu-Gly-Leu-Dpa-Ala-Arg-NH2·TFA [where Mca is (7-methoxycoumarin-4-yl)acetyl, Dpa is N-3-(2,4-dinitrophenyl)-L-2,3-diaminopropionyl, and TFA is trifluoroacetic acid] (Merck Millipore, Darmstadt, Germany) was added to the reaction at a final concentration of 7.5 μM, and the fluorescence was monitored with 340/30 excitation and 400/30 emission filters, using a Synergy 2 plate reader (BioTek, Winooski, VT) at 37° C. The reactions were followed spectroscopically for 120 min and initial rates were determined from the linear portion of the increase in fluorescence signal due to release of the fluorescent MCA group. The tight binding equation, derived from the Morison equation, was used to calculate the dissociation constant (*K*_*i*_):

$$\frac{V_{o}-V_{i}}{V_{i}}=\frac{\left[I_{o}\right]}{K_{i}\left(1+\frac{\left[S_{o}\right]}{K_{m}}\right)}$$ (*Eq*.1)

where Vi is the steady-state rate in the presence of the inhibitor, V_0_ is the steady-state rate in the absence of the inhibitor, K_m_ is the Michaelis constant for substrate cleavage, and [S_0_] and [I_0_] are the initial concentrations of the substrate and inhibitor, respectively. *K*_*i*_ was calculated by plotting the initial velocities against different concentrations of the inhibitors. The inhibition constants reported are the average values obtained from three independent experiments, and the error bars represent the standard error of the mean (SEM). Calculations were performed using *K*_*m*_ values of 9.395 μM for MMP-9_CAT_, as determined from at least three Michaelis–Menten kinetic experiments in our laboratory.

### ELISA for IL-17A binding

ELISA plates (Greiner Bio-One, Kremsmünster, Austria) were incubated with 100 µl of 0.5 µg/µl goat anti-hIL-17A antibodies (R&D Systems, Minneapolis, MN) for 1 h, washed with PBS supplemented with 0.05% Tween-80 (PBST), and added with 100 µl of 0.35 µg/µl IL-17A (PeproTech, Rocky Hill, NJ) for an additional hour. The plates were then washed with PBST and blocked by incubation with 100 µl of PBS supplemented with 3% skin milk for 1 h. After blocking, the plates were washed, incubated with either HD_N-TIMP2,V3_, N-TIMP2, or V3, and shaken for 1 h. The plates were then washed with PBST, incubated for 1 h with 100 µl of 0.05 µg/µl goat anti-hIL-17RA antibodies (R&D Systems), and then incubated with secondary peroxidase-conjugated streptavidin (Jackson ImmunoResearch, 1:10000 dilution). Finally, 100 µl of a horseradish peroxidase (HRP) chromogenic 3, 3', 5, 5' tetramethyl benzidine (TMB) substrate solution (Dako, Santa Clara, CA) were added to the plates. The reaction was stopped by adding 1 M sulfuric acid, and it was analyzed using a Tecan Infinite M200 plate reader at 450 nm. Data shown are the average of a triplicate experiment, where error bars represent the SEM. Statistical analyses were performed using Student's *t*-test.

### Binding assays using the octet red system

Binding assays were conducted using bio-layer interferometry by the Octet Red system (ForteBio, Fremont, CA). All steps were performed at 30° C with shaking at 1500 rpm in a 96-well plate (200 μl per well). For *K*_*D*_ measurements, the Protein A sensors were loaded with 10 μg/ml of either HD_N-TIMP2,V3_ or V3 for 300 s (the loaded protein did not saturate the sensor), followed by washing in PBS supplemented with 1% BSA and 0.05% Tween. Then, the sensors were reacted for 300 s (association phase) with increasing concentrations of the target protein (MMP-9 or IL-17A) and moved to wells that contained the buffer (PBS, 1% BSA, 0.05% Tween) for another 1800–2500 s (dissociation phase). Association and dissociation were measured as changes in light interference over time, and curves are presented after subtracting parallel measurements from unloaded sensors. Sensorgrams were fitted with a 1:1 binding model using the Octet data analysis software (version 8.1), and the *K*_*D*_ values were determined. For the simultaneous binding experiments, Protein A sensors were loaded with 10 μg/ml of either HD_N-TIMP2,V3_ or V3 for 300 s, and then washed in buffer (PBS, 1% BSA, 0.05% Tween). Next, the sensors were reacted with the first target, IL-17A (10 μg/ml), and then with the second target, MMP-9_CAT_ (15 μg/ml). The association phase lasted 180 s and the washing step (dissociation phase) lasted 30 s.

### Cell cultures

Cells from the MDA-MB-231 human breast cancer cell line (kindly provided by Prof. Smadar Cohen, Ben-Gurion University of the Negev, Israel) were maintained in an RPMI-1640 medium (Biological Industries, Cromwell, CT) supplemented with 10% FBS (Thermo Fisher Scientific), 1% L-glutamine (Biological Industries), and 1% penicillin/streptomycin (Biological Industries). Cells from the CCD-1070Sk normal human skin fibroblast cell line (ATCC) were maintained in an EMEM medium (Biological Industries) supplemented with 10% FBS, 1% L-glutamine, and 1% penicillin/streptomycin.

### Inhibition of IL-17A-induced Gro-α secretion in fibroblasts

The normal human skin fibroblast cell line CCD-1070Sk was added to a 96-well plate (Thermo Fisher Scientific), such that each well contained 1 × 10^4^ cells. The plates were incubated for 24 h at 37° C in 5% CO_2_ until 95%–100% confluency was reached, and they were then stimulated for additional 24 h with 10 ng/ml IL-17A (PeproTech) in the presence of HD_N-TIMP2,V3_ or the mono-specific controls. The cell culture medium was then collected for Gro-α analysis, and Gro-α in culture supernatants was measured using an ELISA kit (PeproTech) according to the manufacturer’s guidelines. Data shown is the average of a triplicate experiment, and error bars represent the SEM. Statistical significance was determined using column statistics and Student’s *t*-tests, with *p* < 0.001 considered statistically significant.

### Expression of IL-17A receptor on MDA-MB-231 cell line

MDA-MB-231 cells were treated with trypsin-EDTA (Biological Industries) and divided in 96-well plate, such that each well contained 1 × 10^5^ cells. The cells were centrifuged at 150 × g for 5 minutes and then washed twice with the buffer assay (200 μl of 50 mM Tris HCl pH 7.5, 5 mM CaCl_2_, 100 mM NaCl and 0.1% BSA). Cellular IL-17A receptor levels were detected by adding 100 μl biotinylated goat anti- IL-17RA (R&D Systems, MN, USA) to each well followed by incubation and shaking at 4° C. The cells were then centrifuged and washed twice with 200 μl buffer assay and incubated with 100 μl of FITC conjugated neutravidin antibody (Thermo Fisher, MA, USA) for 30 minutes at 4° C. The cells were then centrifuged and washed twice with 200 μl of buffer assay and were analyzed using Accuri C6 (BD Biosciences, CA, USA) and FlowJo software (Tree star Inc. OR, USA).

### Boyden chamber invasion assay

An *in-vitro* Boyden chamber assay was performed using ThinCert™ 24-well inserts (Greiner Bio-One) as previously described, with minor modifications [62]. The ThinCert™ cell culture insert membranes were coated with Matrigel^®^, diluted 1:30 in an RPMI medium without serum in the upper chamber compartment (30 µg per chamber compartment [63–65]). The lower compartment of the chamber was filled with 600 μl RPMI. Then, 3.5 × 10^4^ MDA-MB-231 cells were treated with 10 ng/ml IL-17A (PeproTech) together with 500 nM of either HD_N-TIMP2,V3_ or the mono-specific variants. The proteins were added to the pre-coated ThinCert™ cell culture inserts, which were then incubated for 6 h at 37° C under 5% CO_2_. Invasive cells were stained with the Dipp Kwik Differential Stain Kit (American MasterTech Scientific, Lodi, CA) and detected using the EVOS FL Cell Imaging System (Thermo Fisher Scientific) at a ×20 magnification. The experiment was performed in a triplicate; in each experiment, 10 fields were counted for each treatment and the average of cells per field was determined. Error bars in figures represent the SEM. Statistical significance was determined by column statistics and Student's *t*-tests, with *p* < 0.005 considered significant.

### Scratch assay

MDA-MB-231 cells (9 × 10^4^ cells) were cultured as confluent monolayers in a 24-well plate, and, after they adhered to the plate surface, were scratched by removing a strip across the well with a p200 pipette tip. The scratched monolayers were then washed twice to remove non-adherent cells. Next, 600 μl of medium containing 30 ng/ml IL-17A (PeproTech) and 10 nM HD_N-TIMP2,V3_ or the mono-specific variants were added. The plate was photographed at the microscopy unit of the NIBN immediately after performing the scratch, and again 24 h later, using Operta (PerkinElmer, Waltham, MA) and Harmony 3.1.1 software. The experiment was performed in a triplicate and the images were analyzed using ImageJ. Error bars in the figures represent the SEM, and statistical significance was determined by column statistics and Student’s *t*-tests, with *p* < 0.05 considered significant.

## SUPPLEMENTARY MATERIALS FIGURES



## Abbreviations

ADAM17: a disintegrin and metalloproteinase 17
IL-17A: interleukin-17A
IL-17RA: interleukin-17A receptor
MMP: matrix metalloproteinase
SEM: standard error of the mean
TIMP2: tissue inhibitor of metalloproteinase 2
TL1A: TNF-like ligand 1A
