# Supplementary material for: A bi-specific inhibitor targeting IL-17A and MMP-9 reduces invasion and motility in MDA-MB-231 cells
Source: Oncotarget. 2018 Jun 19;9(47):28500–13. doi: 10.18632/oncotarget.25526 (PMC6033355; doi:10.18632/oncotarget.25526)
Supplement: Supplementary file 1 [file oncotarget-09-28500-s001.pdf]

A bi-specific inhibitor targeting IL-17A and MMP-9 reduces invasion and motility in MDA-MB-231 cells

SUPPLEMENTARY MATERIALS

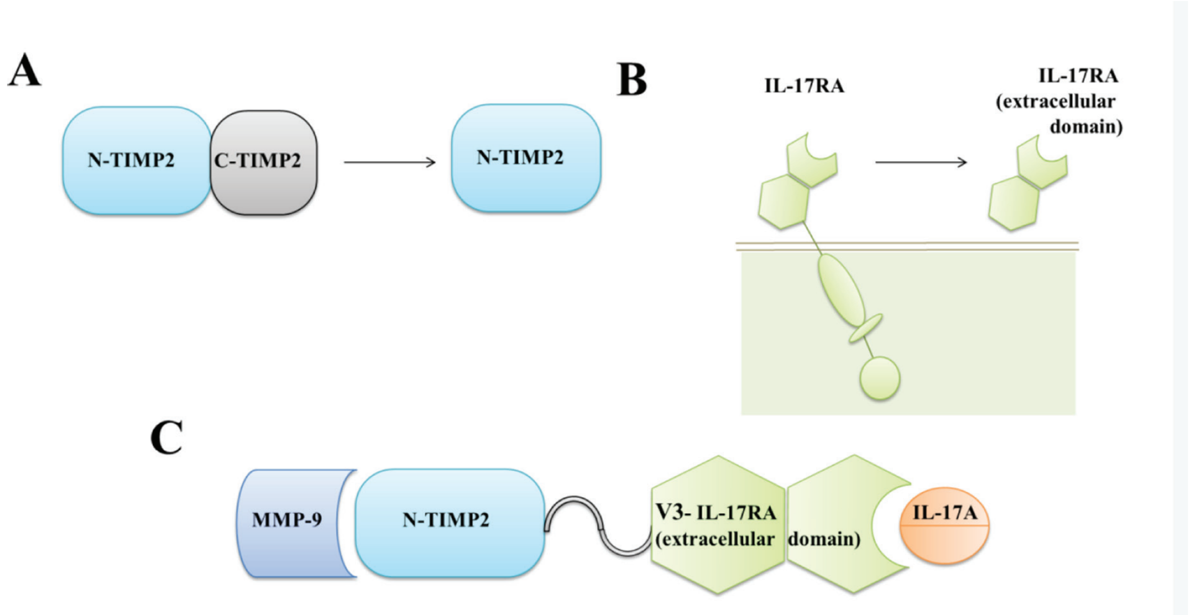

Supplementary Figure 1: Mono- and bi-specific inhibitors used in this study. (**A**) The TIMP2 protein, comprising an N and a C domain. In this work, we used only the N domain of TIMP2 (N-TIMP2). (**B**) We used only the extracellular domain of the IL-17A receptor. (**C**) The bi-specific inhibitor $HD_{N\text{-}TIMP2,V3}$, comprising both N-TIMP2 and the V3 soluble extracellular domain of the IL-17A receptor, thus simultaneously binding both MMP-9 and IL-17A.

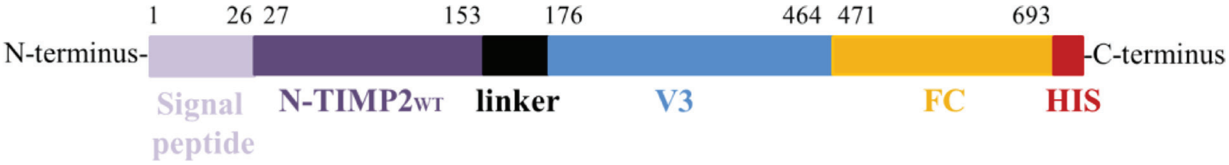

Supplementary Figure 2: The HD$_{N\text{-}TIMP2,V3}$ construct. The construct includes the TIMP2 natural leader peptide (residues 1-26), N-TIMP2$_{WT}$ (residues 27-153), a flexible linker (SGGGGSGGGGSGGGGS), the V3 soluble extracellular domain of the IL-17A receptor (residues 176-464), a human IgG1 Fc (residues 471-693), and a 6×His tag.

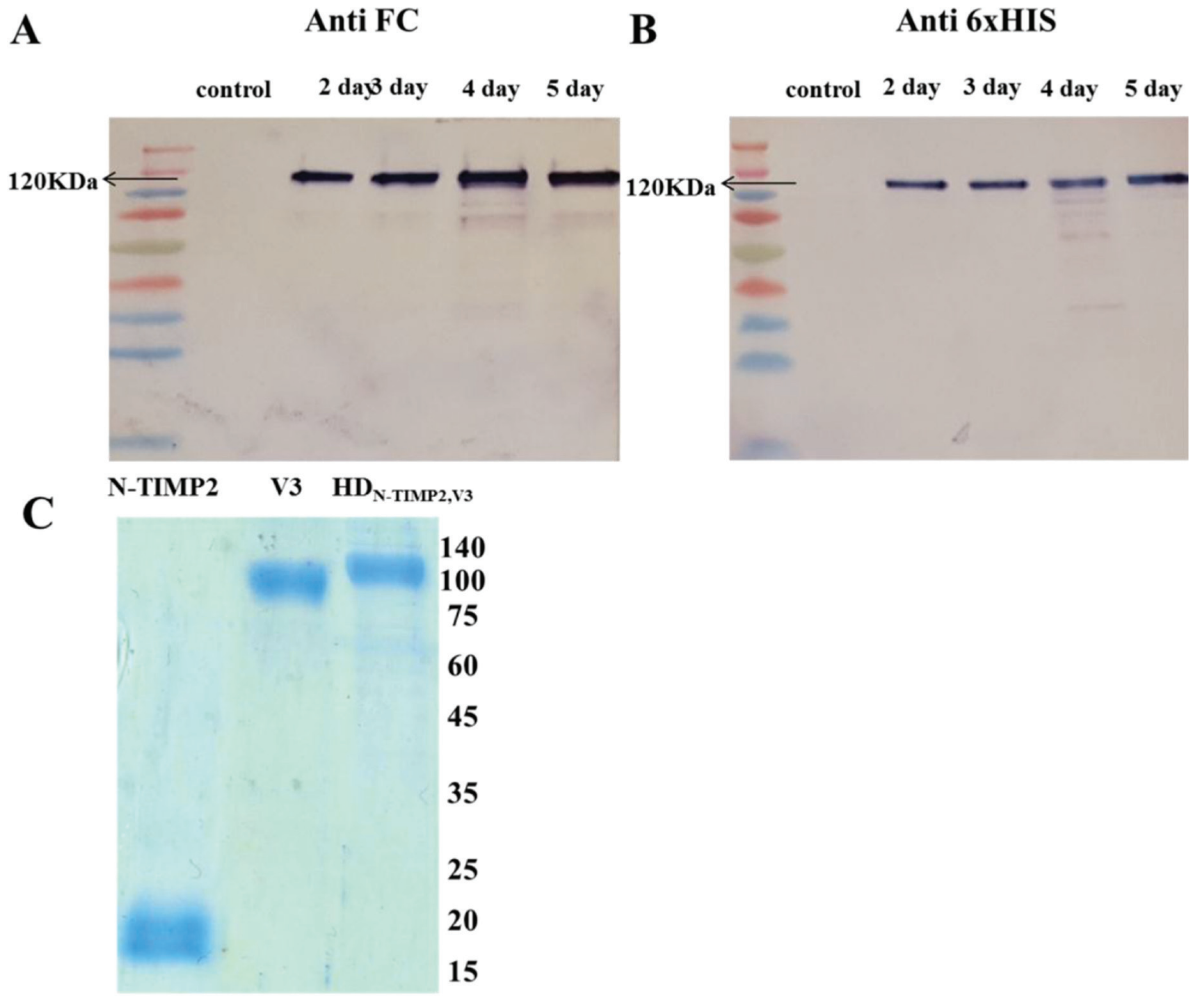

Supplementary Figure 3: Production and purification of HD$_{N\text{-}TIMP2,V3}$ and of the mono-specific controls. (**A**, **B**) Western blot for the FC domain (A) and for the 6×His tag (B) over five days from transfection in HEK293 cells. Control: transfected HEK293 cells. (**C**) SDS-PAGE of the purified proteins following an affinity column chromatography.

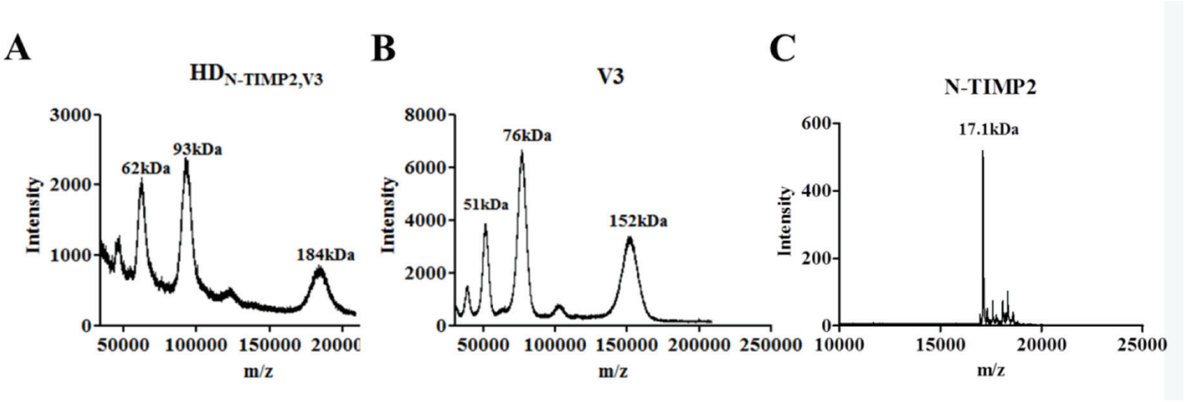

Supplementary Figure 4: Mass spectrometry analysis of $HD_{N\text{-}TIMP2,V3}$ (**A**), V3 (**B**), and N-TIMP2 (**C**). $HD_{N\text{-}TIMP2,V3}$ and V3 show several peaks: the most intense peaks at the highest mass-to-charge ratio, (i.e., 93 kDa and 76 kDa in panels A and B, respectively), represent the single-charged molecular ion; the two lower-intensity peaks preceding it (i.e., 62 kDa and 51 kDa in panels A and B, respectively), correspond to the double- and triple-charged molecular ions [63]; and the last and most intense peaks (184 kDa and 152 kDa in panels A and B, respectively), represent dimers.

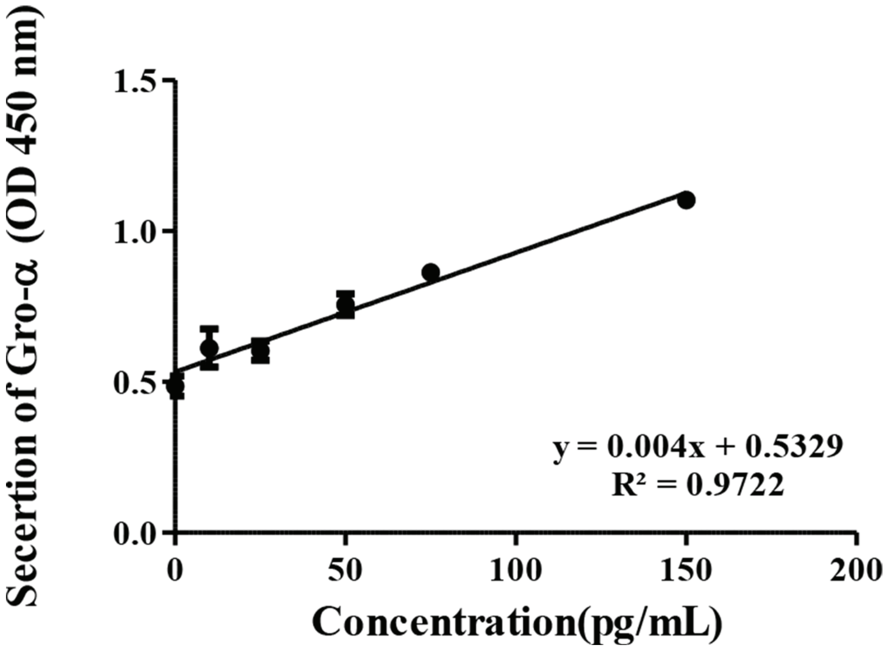

Supplementary Figure 5: Gro-α calibration curve. Six concentrations of commercially available Gro-α were used and the secretion of Gro-α was examined in an ELISA.

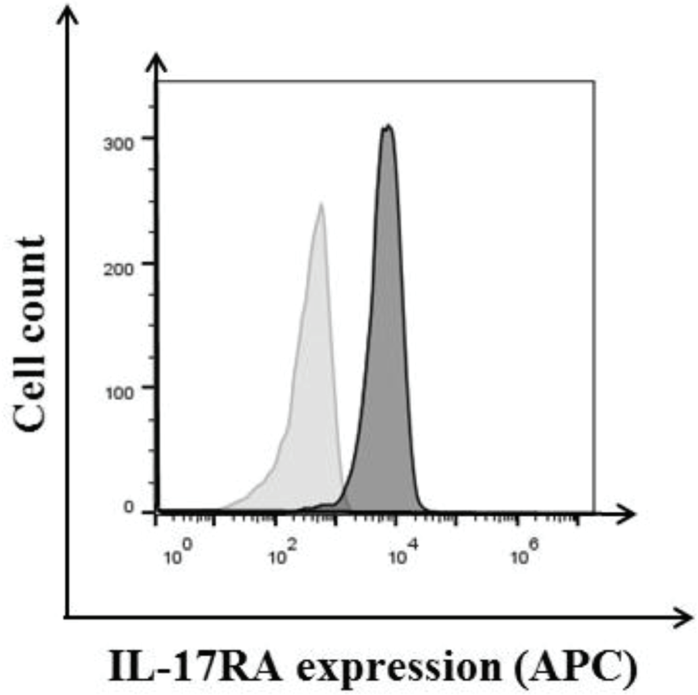

Supplementary Figure 6: Expression of IL-17A receptor (IL-17RA) on MDA-MB-231 cell lines (FACS analysis results). Grey: cells only; black: IL-17A expression.

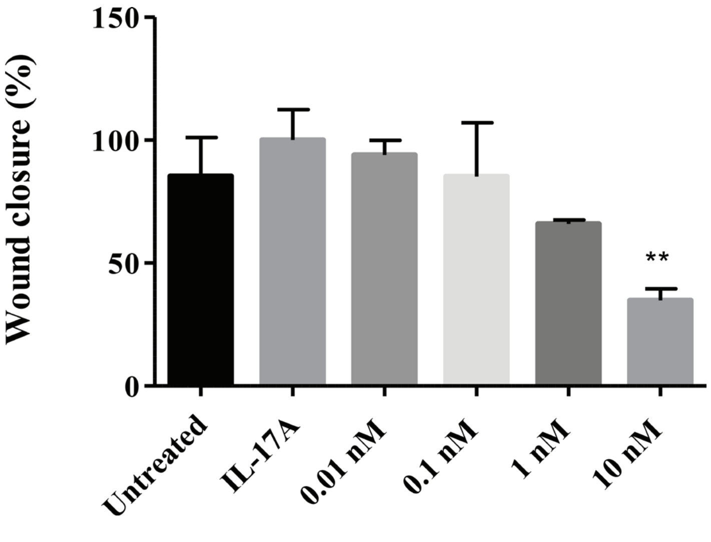

Supplementary Figure 7: Inhibition of the migration of MDA-MB-231 cells by $HD_{N-TIMP2,V3}$ in a scratch assay. Cells were treated with IL-17A together with various concentrations of $HD_{N-TIMP2,V3}$ (0.01–10 nM, as indicated). The quantification of migrated cells is normalized to cells treated with IL-17A alone. Bars represent the average (± SEM) of a triplicate experiment. $^{**}p < 0.01$ (Student's *t*-test, as compared with cells treated with IL-17A alone).
